# Incidence of pneumonitis following the use of different anaplastic lymphoma kinase tyrosine kinase inhibitor regimens: An updated systematic review and meta‐analysis

**DOI:** 10.1002/cam4.5913

**Published:** 2023-04-05

**Authors:** Wenting Qie, Qian Zhao, Linlin Yang, Bing Zou, Yanan Duan, YueYuan Yao, Linlin Wang

**Affiliations:** ^1^ Department of Oncology Weifang Medical University Weifang Shandong People's Republic of China; ^2^ Department of Radiation Oncology, Shandong Cancer Hospital and Institute Shandong First Medical University and Shandong Academy of Medical Sciences Jinan Shandong People's Republic of China; ^3^ Department of Oncology Renmin Hospital of Wuhan University Wuhan People's Republic of China

**Keywords:** ALK TKIs, lung toxicity, meta‐analysis, NSCLC, pneumonitis

## Abstract

**Objectives:**

Anaplastic lymphoma kinase tyrosine kinase inhibitors (ALK TKIs) have shown remarkable clinical activity in patients with non‐small‐cell lung cancer (NSCLC). However, pneumonitis is a serious side effect of ALK TKIs in NSCLC patients. In this meta‐analysis, we aimed to determine the incidence of ALK‐TKI‐associated pneumonitis.

**Materials and Methods:**

We searched electronic databases to identify relevant studies published until August 2022. The incidence of pneumonitis was calculated using a fixed‐effects model when no substantial heterogeneity was observed. Otherwise, a random‐effects model was used. Subgroup analyses of different treatment groups were performed. Statistical analyses were conducted using STATA 17.0.

**Results:**

Twenty‐six clinical trials involving 4752 patients were eligible for analysis. All‐grade pneumonitis incidence was 2.92% (95% confidence interval [CI]: 1.79%–4.27%), high‐grade (Grade 3–4) pneumonitis incidence was 1.42% (95% CI: 0.84%–2.12%) and Grade 5 pneumonitis incidence was 0.09% (95% CI: 0.00%–0.28%). The subgroup analysis showed that brigatinib was associated with the highest incidence of both all‐grade and high‐grade pneumonitis (7.09% and 3.06%, respectively). ALK TKI treatment after chemotherapy was associated with a higher incidence of all‐grade and high‐grade pneumonitis than first‐line ALK TKI treatment (7.73% vs. 2.26% and 3.64% vs. 1.26%, respectively). Cohorts from Japanese trials had a higher incidence of all‐grade and high‐grade pneumonitis.

**Conclusion:**

Our study provides precise data on the incidence of pneumonitis in patients receiving treatment with ALK TKIs. Overall, ALK TKIs have tolerable pulmonary toxicity. Early pneumonitis identification and treatment are required to prevent further deterioration in patients receiving treatment with brigatinib and in those who received prior chemotherapy, particularly in the Japanese population.

## Graphical abstract

Incidence of pneumonitis was 2.92% in NSCLC patients treated with ALK TKIs. Patients from Japan had a increased risk of developing pneumonitis. Prior chemotherapy increases the incidence of pneumonitis, and is more likely to occur with brigatinib than other ALK TKIs.

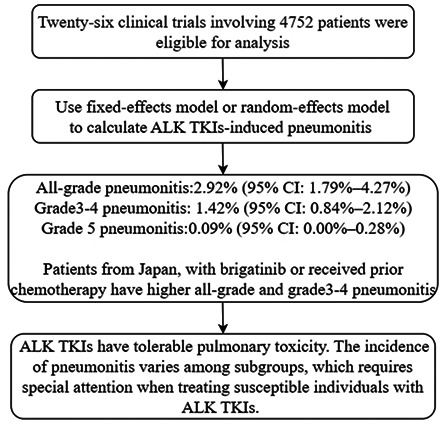

## 1 INTRODUCTION

Since the Food and Drug Administration (FDA) approved the use of the first‐generation anaplastic lymphoma kinase tyrosine kinase inhibitor (ALK TKI), crizotinib, in 2011,^1^ several second‐ and third‐generation ALK TKIs, including alectinib, ceritinib, brigatinib and lorlatinib, were developed. These five ALK TKIs are FDA‐approved medications for first‐line treatment.^2^, ^3^, ^4^, ^5^ ALK TKI treatment has resulted in a 16.6‐ to 34.8‐month prolongation of median progression‐free survival (PFS), correspondingly improving the 5‐year overall survival (OS) rate by up to 62.5%^6^, ^7^ and the objective response rates of this treatment range from 67.7% to 87.5%; these findings indicate excellent therapeutic efficiency against ALK rearrangements.^8^, ^9^

However, adverse events (AEs) associated with ALK TKIs inevitably occur and cannot be ignored. Toxicity profiles, such as visual abnormalities, nervous lesions, hyperlipidaemia and peripheral oedema, have been observed to be associated with ALK TKIs.^10^, ^11^ The incidence of serious adverse events (SAEs) ranges from 27.1% to 45.7% in patients receiving treatment with ALK TKIs.^12^, ^13^

Among these SAEs, pneumonitis is a rare but hazardous AE that can be life threatening and its incidence has gradually increased in recent years. Currently available data regarding the incidence of pneumonitis do not seem conclusive. The incidence of pneumonitis varies from 0.4% to 9% among different studies.^14^, ^15^ Once pneumonitis occurs, dose reduction, treatment suspension or medication discontinuation is required, which can adversely affect OS and curative intentions.^16^ Timely diagnosis and early treatment of pneumonitis caused by ALK‐TKIs are key to improving the prognosis of these patients. Therefore, standardised diagnosis and effective treatment of ALK‐TKI‐induced pneumonitis are clinical concerns that have attracted increasing attention from clinicians worldwide.

An earlier systematic review and meta‐analysis of ALK‐TKI‐induced pneumonitis was published in 2019.^17^ As treatment strategies for patients with ALK‐rearrangement non‐small‐cell lung cancer (NSCLC) are constantly evolving and given the continuing concerns regarding ALK‐TKI‐induced pneumonitis, we aimed to update this previous meta‐analysis.

## 2 METHODS

This meta‐analysis was performed in accordance with the Preferred Reporting Items for Systematic Reviews and Meta‐Analyses (PRISMA) guidelines.^18^

### 2.1 Data sources and searches

Searches were conducted in electronic databases, including PubMed, Web of Science, Embase and Cochrane Library, for relevant articles published until August 2022. The following search term, which combined synonyms using the keywords, “crizotinib,” “brigatinib,” “lorlatinib,” “ceritinib,” “alectinib,” and “lung cancer,” was used: (crizotinib OR brigatinib OR lorlatinib OR ceritinib OR alectinib) AND (lung cancer). These five ALK TKIs were chosen for the study because at the time of the study, the FDA had approved these drugs for the treatment of NSCLC with ALK rearrangement.

### 2.2 Study selection and data extraction

The inclusion criteria were as follows: (1) studies involving patients with advanced NSCLC who were receiving approved doses of ALK TKIs in clinical trials and (2) studies providing detailed information on ALK‐TKI‐associated pneumonitis. Conference abstracts, brief reports, case reports, reviews and non‐English articles were excluded. Studies involving neoadjuvant therapy or the use of ALK‐TKIs in conjunction with other anticancer treatment strategies were excluded. In case different studies involved the same population, the most recent and relevant article was chosen for our meta‐analysis.

Two investigators independently assessed all the trials and collected the following information from the identified articles: initial author's name, publication year, region and treatment line. The treatment lines were divided as follows: (1) first‐line ALK TKI therapy without any prior systemic therapy, (2) ALK TKI therapy following prior chemotherapy without any prior ALK TKI therapy and (3) ALK TKI therapy following prior chemotherapy or other ALK TKI therapy (previously treated group). The details of all‐grade, high‐grade (Grade 3–4) and Grade 5 pneumonitis‐related deaths were collected. In addition, the number of patients with cough and dyspnoea was recorded.

### 2.3 Quality assessment and statistical analysis

The Newcastle‐Ottawa Scale (NOS) was used to evaluate the quality of the included studies, and studies with an NOS score ≥6 were considered to be of high quality.^19^ We used a systematic analytical approach to compute the pooled pneumonitis rates in all the eligible studies. The weights of each study were determined using a random‐effects model directly from the STATA ‘metaprop’ command. Before pooling, the variances were stabilised using the Freeman–Tukey double arcsine transformation method.^20^ Heterogeneity was evaluated using the *Q* statistic and Higgins inconsistency index (*I* ^2^) test. A *p‐*value of 0.05 was used to assess the test's statistical significance. An *I* ^2^ greater than 50% was considered to represent a large amount of heterogeneity, in which case the source of heterogeneity was explored. The potential causes of heterogeneity were investigated using subgroup analyses and meta‐regression. All statistical analyses were performed using Stata version 17.0.

## 3 RESULTS

### 3.1 Eligible studies and characteristics

**FIGURE 1:**
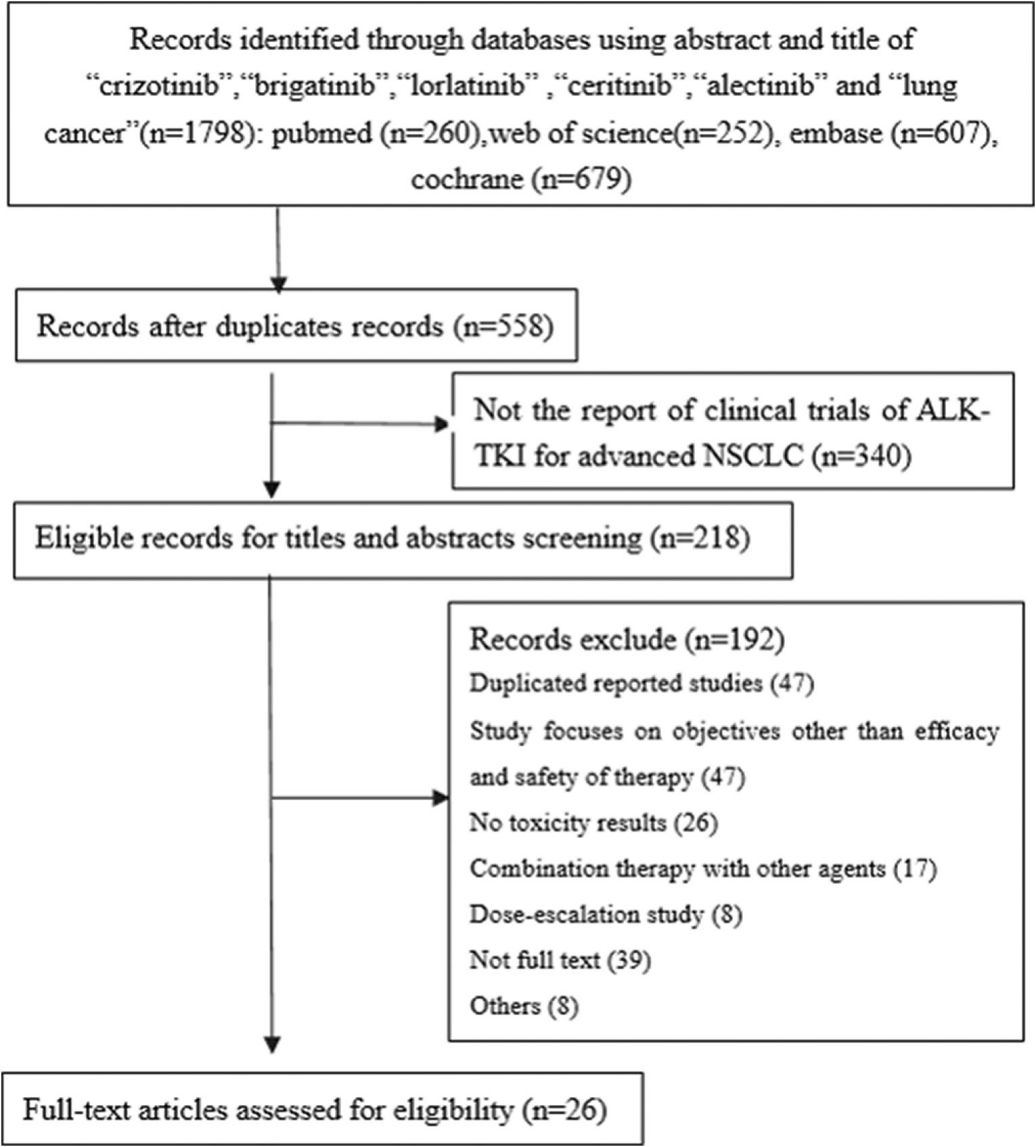
Flow diagram of study inclusion.

Our literature search yielded a total of 1798 articles, of which 1680 were excluded after the initial screening of abstracts or titles (Figure 1). After screening and assessing eligibility, 26 studies, which included 31 cohorts, were selected.^6^, ^9^, ^10^, ^12^, ^14^, ^15^, ^16^, ^21^, ^22^, ^23^, ^24^, ^25^, ^26^, ^27^, ^28^, ^29^, ^30^, ^31^, ^32^, ^33^, ^34^, ^35^, ^36^, ^37^, ^38^, ^39^

Collectively, the included trials involved a total of 4752 unique patients. The features and details of the quality assessments of the included studies are presented in Table 1. The study sample sizes ranged from 11 to 1066 patients. The baseline Eastern Cooperative Oncology Group performance status of all the patients ranged from 0 to 2. The drugs tested in these cohorts included crizotinib (10 cohorts, 2258 patients), ceritinib (7 cohorts, 831 patients), alectinib (6 cohorts, 769 patients), brigatinib (4 cohorts, 338 patients) and lorlatinib (4 cohorts, 556 patients). Five studies recruited subjects from Japan, 15 from other individual countries and 11 from multiple countries including Japan. Twelve of the cohorts were treated with first‐line ALK TKIs, three cohorts had received prior chemotherapy and 16 cohorts had received prior chemotherapy and ALK‐TKI treatment (previously treated group). The NOS scores of the included studies ranged from 6 to 8.

**TABLE 1:** Baseline characteristics of 26 included trials.

				No. (%) of patients			
Authors	Drug	Phase	No. of treated patients	All‐grade pneumonitis	Grade 3–4 pneumonitis	Pneumonitis‐related death	Newcastle‐Ottawa‐Scale (NOS) evaluation
Camidge D. R.^16^	Brigatinib	III	137	7	4	0	8
	Crizotinib	III	138	3	1	0	
Shaw A.T.^10^	Lorlatinib	III	149	2	0	1	8
	Crizotinib	III	147	2	1	0	
Sai‐Hong Ignatius Ou^22^	Alectinib	II	225	1	1	0	6
Yilong Wu^23^	Ceritinib	I/II	103	NA	1	1	7
Caicun Zhou^24^	Alectinib	III	187	NA	NA	0	8
	Crizotinib	III	62	NA	NA	3	
T. Mok^6^	Alectinib	III	152	NA	0	0	6
	Crizotinib	III	151	NA	3	1	
J Wolf^36^	Alectinib	III	79	NA	2	NA	8
Benjamin J Solomon^25^	Crizotinib	III	172	NA	4	1	8
Yi‐Long Wu^9^	Crizotinib	III	104	NA	3	1	8
Sun Min Lim^37^	Ceritinib	II	32	4	2	2	7
Jean‐Charles Soria^26^	Ceritinib	III	189	4	NA	1	6
Lucio Crinò^12^	Ceritinib	II	140	2	1	0	7
Dong‐Wan Kim^14^	Ceritinib	I	246	8	7	1	8
Fiona Blackhall^27^	Crizotinib	II	1066	25	6	4	6
D Ross Camidge^38^	Crizotinib	I	149	NA	3	0	8
Alice T. Shaw^39^	Crizotinib	III	173	NA	6	2	8
Rafal Dziadziuszko^28^	Alectinib	II	87	NA	2	0	7
Ibiayi Dagogo‐Jack^29^	Lorlatinib	II	23	NA	NA	1	6
Rudolf M. Huber^15^	Brigatinib	II	110	10	4	NA	7
Benjamin J Solomon^30^	Lorlatinib	II	275	3	3	0	8
Katsuyuki Kiura^31^	Ceritinib	III	11	0	0	NA	7
ThomasE Stinchcombe^32^	Brigatinib	II	20	NA	2	NA	7
Shun Lu^33^	Lorlatinib	II	109	NA	NA	3	7
Makoto Nishio^34^	Brigatinib	II	72	6	2	0	7
Toyoaki Hida^21^	Alectinib	III	103	8	5	NA	8
	Crizotinib	III	104	8	3	NA	
Hiroyasu Kaneda^35^	Ceritinib	II	110	2	1	NA	7

### 3.2 Incidence of all‐grade pneumonitis

**FIGURE 2:**
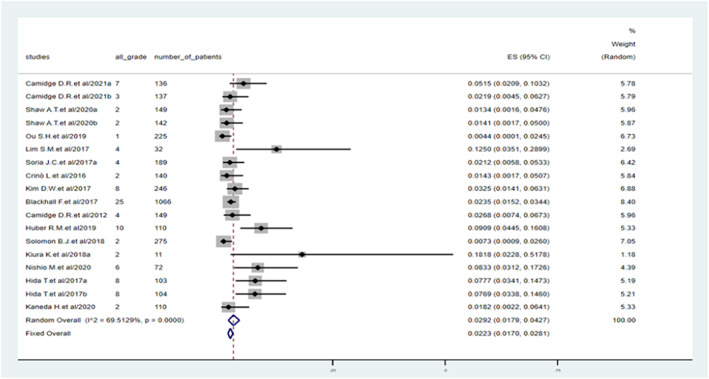
Incidence of all‐grade pneumonitis during treatment with anaplastic lymphoma kinase tyrosine kinase inhibitors.

All‐grade pneumonitis developed in 100 of 3396 ALK TKI‐treated patients, with an incidence of 2.92% (95% confidence interval [CI]: 1.79%–4.27%). The predicted incidence of all‐grade pneumonitis ranged from 0.9% (95% CI: 0.14%–2.12%) with lorlatinib to 7.09% (95% CI: 4.44%–10.26%) with brigatinib (Figure 2). There was heterogeneity in the incidence of all‐grade pneumonitis among the studies (*I* ^2^ = 69.5%).

The subgroup analysis also showed that the cohorts in the Japanese studies (5.93%, 95% CI: 2.52%–10.40%) had a higher incidence than those in the non‐Japanese studies and in studies in multiple countries including Japan (3.00% and 2.12%, respectively) (Table 2); however, the difference was not significant (*p* = 0.09). Furthermore, patients who received chemotherapy prior to treatment with ALK TKIs were more likely to develop all‐grade pneumonitis (7.73%, 95% CI: 4.39%–11.85%) compared with those who received first‐line ALK TKI treatment (2.26%, 95% CI: 1.23%–3.54%) and previously treated group (2.68%, 95% CI: 1.23%–4.55%); however, the difference was not significant (*p* = 0.21) (Supplementary Table 1). A similar incidence of all‐grade pneumonitis was reported in I/II/III phase studies.

**TABLE 2:** Results of multiple subgroup analysis for the incidence of all‐grade pneumonitis.

Subgroup	No. of patients	No. of studies	Incidence (%) (95% CI)	Heterogeneity test	
				*I* ^2^	Q test *p‐*value
ALK‐TKI					
Crizotinib	1598	5	2.68 (1.42–4.28)	46.3%	0.114
Alectinib	328	2	1.80 (0.54–3.64)	–	–
Ceritinib	728	6	2.50 (0.69–5.07)	55.1%	0.049
Brigatinib	318	3	7.09 (4.44–10.26)	–	–
Lorlatinib	424	2	0.90 (0.14–2.12)	–	–
Treatment line					
First‐line	753	5	2.26 (1.23–3.54)	5.0%	0.378
Prior chemotherapy	207	2	7.73 (4.39–11.85)	–	–
Previously treated	2436	11	2.68 (1.23–4.55)	74.1%	<0.001
Country					
Japan plus other countries	2220	8	2.12 (1.14–3.34)	56.5%	0.024
Japan study	400	5	5.93 (2.52–10.40)	53.8%	0.070
Non‐Japan study	776	5	3.00 (0.82–6.22)	74.3%	0.004
Generation					
First‐generation TKI	1598	5	2.68 (1.42–4.28)	46.3%	0.114
Second‐generation TKI	1374	11	3.92 (1.88–6.53)	73.8%	<0.001
Third‐generation TKI	424	2	0.90 (0.14–2.12)	–	–
Study phase					
I	395	2	3.02 (1.49–5.00)	–	–
II	971	8	2.79 (1.10–5.10)	78.8%	<0.001
III	2030	8	3.28 (1.45–5.67)	62.4%	0.010

Abbreviations: ALK TKI, anaplastic lymphoma kinase tyrosine kinase inhibitor; CI, confidence interval.

### 3.3 Incidence of high‐grade pneumonitis

**FIGURE 3:**
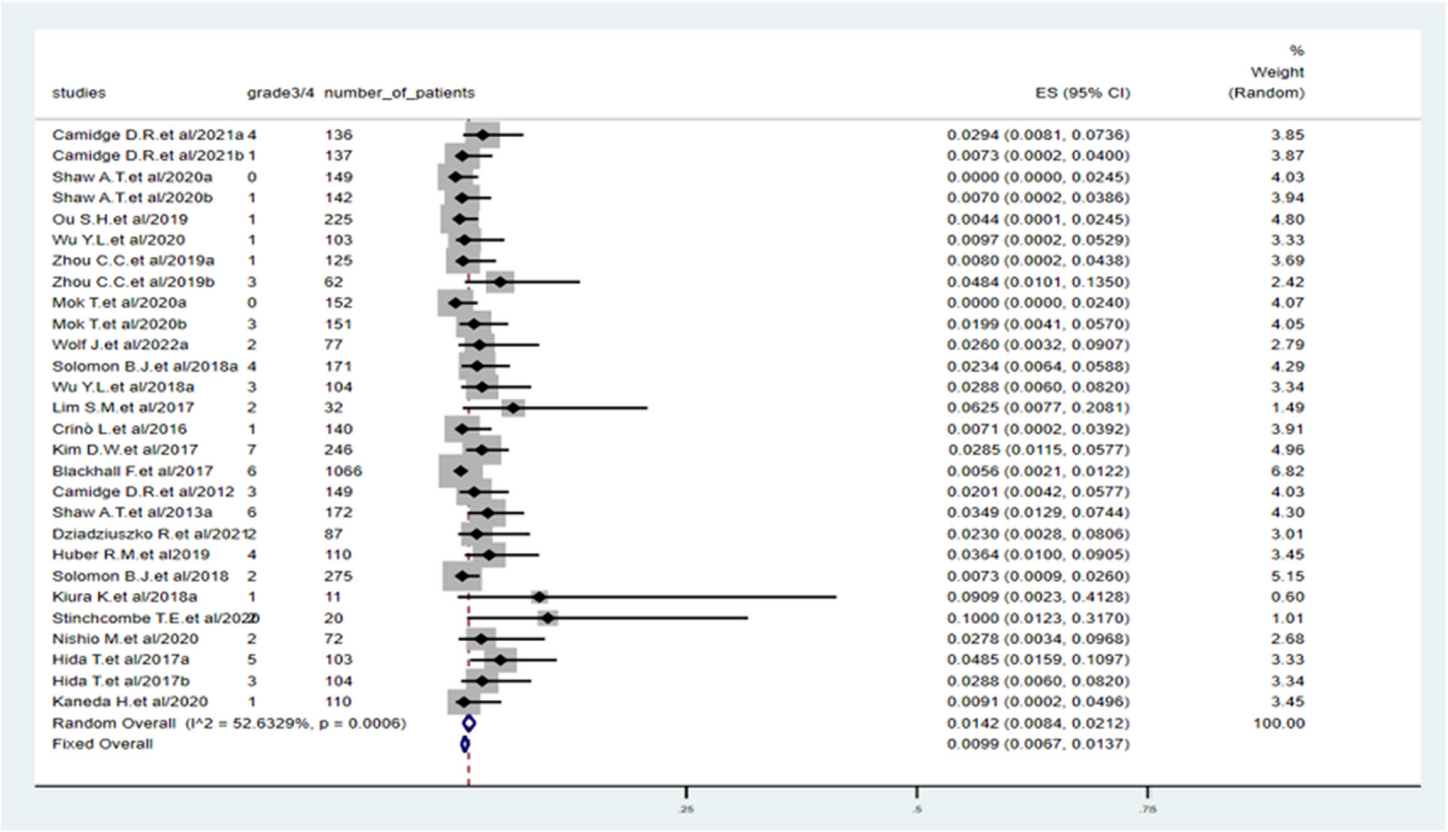
Incidence of high‐grade pneumonitis during treatment with anaplastic lymphoma kinase tyrosine kinase inhibitors.

Across all the study cohorts, 71 of 4431 patients had high‐grade pneumonitis. The incidence of Grade 3 and higher‐grade pneumonitis in the 24 cohorts is shown in Figure 3. The pooled incidence was 1.42% (95% CI: 0.84%–2.12%). Meanwhile, the heterogeneity test revealed heterogeneity in all the results (*I* ^2^ = 52.6%).

According to the subgroup analysis, the incidence of high‐grade pneumonitis related to second‐generation ALK TKIs, including alectinib, ceritinib and brigatinib (1.54%, 95% CI: 0.68%–2.64%), was similar to that related to the first‐generation ALK TKI, crizotinib (1.76%, 95% CI: 0.85%–2.93%), but it was higher than that related to the third‐generation ALK TKI, lorlatinib (0.35%, 95% CI: 0.00%–1.27%). On comparing different second‐generation ALK TKIs, patients who were administered brigatinib (3.06%, 95% CI: 1.28%–5.41%) had a higher risk of high‐grade pneumonitis than those administered ceritinib (1.18%, 95% CI: 0.15%–2.80%) and alectinib (1.22%, 95% CI: 0.16%–2.98%) (Table 3). High‐grade pneumonitis occurred in 2.20% (95% CI: 0.67%–4.31%) and 1.53% (95% CI: 0.66%–2.67%) of patients in the Japanese and non‐Japanese studies, respectively. However, these differences were not significant (Supplementary Table 2). A similar incidence of high‐grade pneumonitis was reported in I/II/III phase studies.

**TABLE 3:** Results of multiple subgroup analysis for the incidence of high‐grade pneumonitis.

Subgroup	No. of patients	No. of studies	Incidence (%) (95% CI)	Heterogeneity test	
				*I* ^2^	Q test *p‐*value
ALK‐TKI					
Crizotinib	2258	10	1.76 (0.85–2.93)	57.1%	0.013
Alectinib	769	6	1.22 (0.16–2.98)	59.7%	0.030
Ceritinib	642	6	1.18 (0.15–2.80)	28.0%	0.225
Brigatinib	338	4	3.06 (1.28–5.41)	0%	0.551
Lorlatinib	424	2	0.35 (0.00–1.27)	–	–
Treatment line					
First‐line	1016	11	1.26 (0.51–2.27)	43.6%	0.060
Prior chemotherapy	379	3	3.64 (1.90–5.86)	–	–
Previously treated	3036	15	1.16 (0.41–2.18)	54.6%	0.007
Country					
Japan plus other countries	2461	10	1.22 (0.52–2.15)	55.9%	0.016
Japan study	400	5	2.20 (0.67–4.31)	9.6%	0.351
Non‐Japan study	1570	13	1.53 (0.66–2.67)	47.1%	0.031
Generation					
First‐generation TKI	2258	10	1.76 (0.85–2.93)	57.1%	0.013
Second‐generation TKI	1749	16	1.54 (0.68–2.64)	46.3%	0.022
Third‐generation TKI	424	2	0.35 (0.00–1.27)	–	–
Study phase					
I	613	3	1.42 (0.84–4.35)	–	–
II	1674	10	1.08 (0.29–2.21)	55.2%	0.018
III	1929	15	1.55 (0.72–2.62)	49.1%	0.017

Abbreviations: ALK TKI, anaplastic lymphoma kinase tyrosine kinase inhibitor; CI, confidence interval.

### 3.4 Incidence of Grade 5 pneumonitis

**FIGURE 4:**
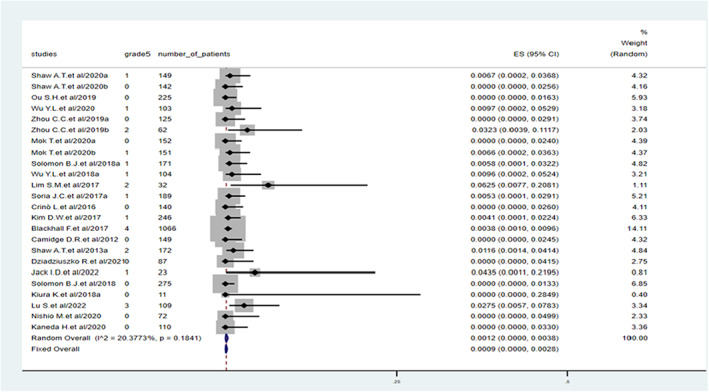
Incidence of Grade 5 pneumonitis during treatment with anaplastic lymphoma kinase tyrosine kinase inhibitors.

The incidence rate of lethal pneumonitis was 0.09% (95% CI: 0.00%–0.28%). The between‐study heterogeneity was low, with an *I* ^2^ of 20.4% (Figure 4).

In the included studies, data on Grade 5 pneumonitis were not available for all the drugs. The likelihood of developing Grade 5 pneumonitis was increased in patients treated with crizotinib (0.30%, 95% CI: 0.06%–0.66%) and lorlatinib (0.18%, 95% CI: 0.00%–0.97%) compared with in those treated with ceritinib (0.03%, 95% CI: 0.00%–0.50%). The incidence of Grade 5 pneumonitis was fourfold higher in the previous chemotherapy group than in the first‐line ALK TKI group (Table 4).

**TABLE 4:** Results of multiple subgroup analysis for the incidence of Grade 5 pneumonitis.

Subgroup	No. of patients	No. of studies	Incidence (%) (95% CI)
ALK‐TKI			
Crizotinib	2017	8	0.30 (0.06–0.66)
Alectinib	589	4	0.00 (NA)
Ceritinib	831	7	0.03 (0.00–0.50)
Brigatinib	72	1	0.00 (NA)
Lorlatinib	556	4	0.18 (0.00–0.97)
Treatment line			
First‐line	1332	10	0.32 (0.03–0.80)
Prior chemotherapy	172	1	1.16 (0.14–4.14)
Previously treated	2561	13	0.00 (0.00–0.13)
Country			
Japan plus other countries	2540	10	0.19 (0.02–0.46)
Japan study	193	3	0.00 (NA)
Non‐Japan study	1332	11	0.33 (0.03–0.85)
Generation			
First‐generation TKI	2017	8	0.30 (0.06–0.66)
Second‐generation TKI	1492	12	0.00 (NA)
Third‐generation TKI	556	4	0.18 (0.00–0.97)
Study phase			
I	395	2	0.18 (0.00–0.28)
II	2242	11	0.02 (0.00–0.23)
III	1428	11	0.21 (0.00–0.67)

Abbreviations: ALK TKI, anaplastic lymphoma kinase tyrosine kinase inhibitor; CI, confidence interval.

### 3.5 Incidence of dyspnoea and cough

The overall incidence of all‐grade dyspnoea was 18.6%. The incidence was similar among the different ALK TKI groups (ranging from 16.63% in the brigatinib group to 20.13% in the crizotinib and lorlatinib groups). However, the probability of all‐grade cough differed markedly among the different ALK TKI groups (Supplementary Figure 1). The highest incidence was observed in the brigatinib group (36.03%), and the lowest, in the lorlatinib group (16.11%).

There was considerable variation in the proportion of patients with high‐grade dyspnoea between the different groups. The incidence of high‐grade dyspnoea in each of the following ALK TKI groups: crizotinib, alectinib, ceritinib, brigatinib and lorlatinib was 2.99%, 4.65%, 4.07%, 3.90% and 0.93%, respectively (Supplementary Figure 2). High‐grade cough was highly unlikely to have occurred. No Grade 5 AEs were related to cough or dyspnoea.

### 3.6 Publication bias

Slight asymmetry was visible in the funnel plots for each grade of pneumonitis, suggesting possible publication bias. The asymmetry of the funnel plots was further evaluated using Egger's test. There was evidence of publication bias based on the results of Egger's test (all *p* < 0.05).

## 4 DISCUSSION

The findings of this meta‐analysis reveal an association between the occurrence of pneumonitis and use of ALK TKIs. In our study, 2.92% of patients receiving treatment with ALK TKIs developed all‐grade pneumonitis, 1.42% developed high‐grade pneumonitis and 0.09% developed Grade 5 pneumonitis. To our knowledge, this is the most thorough systematic review and meta‐analysis of the incidence of pneumonitis following an FDA‐approved ALK TKI regimen in patients with ALK rearrangement in advanced NSCLC.

Suh et al. reported an all‐grade pneumonitis incidence of 2.14%, a high‐grade pneumonitis incidence of 1.33% and a Grade 5 pneumonitis incidence of 0.22%^17^; in comparison, we identified a slightly higher rate of all‐grade pneumonitis. Our findings related to high‐grade pneumonitis are consistent with those of previous meta‐analyses. However, the proportion of patients with Grade 5 pneumonitis has decreased. One potential reason for this proportional redistribution may be the recent focus on pneumonitis as a rare but serious side effect of ALK TKIs and the increasingly prompt management of this condition.

Our study has revealed for the first time that the lowest rate of all‐grade and high‐grade pneumonitis is associated with lorlatinib compared with other ALK TKIs. A single‐arm meta‐analysis showed that lorlatinib had the lowest discontinuation rate (3%) compared with that of alectinib (7%), brigatinib (7%), ceritinib (8%) and crizotinib (8%).^40^ This finding illustrates the pulmonary safety of lorlatinib particularly well. Brigatinib, a second‐generation ALK TKI, was associated with the highest rate of pneumonitis occurrence. The ALTA study reported on the safety of brigatinib for treating crizotinib‐refractory ALK positive NSCLC. All deaths occurred in the groups receiving 90 mg quaque die (QD), 120 mg QD and 180 mg QD and they occurred within 7 days of starting treatment; further, dose interruptions were required in a larger number of patients in the higher dose group. Therefore, a regimen involving run‐in dose escalation from a small to a regular dose was developed. It has been presumed that the occurrence of pneumonitis is related to the dose and usage of treatment and that dose adjustment can control the occurrence of pneumonitis.^15^ However, pneumonitis occurred relatively late with some other ALK TKIs. Crizotinib‐induced pneumonitis has two clinical features. The first type is a severe and irreversible form of pneumonitis that develops early (usually within a month after the initial treatment) and with more severe symptoms. The second type is a late‐onset form that typically occurs around 3 months after starting crizotinib.^41^, ^42^ Median time to lorlatinib‐associated pneumonitis was 73.5 days.^43^ However, there are currently no published data regarding the average time to pneumonitis associated with alectinib and ceritinib. It is important not to overlook pneumonitis due to its late onset.

Chemotherapy remains the key treatment option for cancer. Therefore, we have presented data on the incidence of pneumonitis in patients who received chemotherapy before ALK TKI administration. We found a high incidence (7.73%) of all‐grade pneumonitis in patients who had received prior chemotherapy. In addition, prior chemotherapy increased the rates of high‐grade and Grade 5 pneumonitis; however, the difference was not significant. Our results are in accordance with the finding that chemotherapeutic agents, such as paclitaxel, also cause lung injuries, with an incidence below 1%.^44^, ^45^ However, a similar phenomenon was not observed in the previously treated group. We speculate that the difference in the number of studies included in each group may have caused heterogeneity and bias. The results of the present study would be more convincing if a larger number of randomized controlled trials (RCTs) could have been included. The mechanism underlying superposed lung damage may be complex. Hence, before receiving treatment with ALK TKIs, patients with a history of chemotherapy should undergo stricter screening in order to decrease the risk of pneumonitis and prevent fatal pneumonitis.

Pneumonitis is thought to affect Japanese patients more frequently than other ethnic groups. This result is in accordance with that of earlier reports.^17^, ^46^ Pneumonitis is also one of the most common SAE associated with epidermal growth factor receptor‐TKIs, with a documented incidence of 1.6%–4.3% in Japanese populations and 0.3%–1.0% in non‐Japanese populations.^47^ Krebs von den Lungen‐6 has been reported to serve as a sensitive serum marker and is now used clinically to detect pneumonitis in Japan.^48^ Moreover, genetic variation in surfactant protein‐D (SP‐D) is a risk factor for pneumonitis development in the Japanese population.^49^, ^50^ The German cohort had significantly higher serum SP‐D levels than did the Japanese cohort.^51^ Further scientific research is needed to determine the causes of the difference in incidence between Japan and the rest of the world. The study from Japan did not find a higher probability of Grade 5 pneumonitis; therefore, we speculate that the high prevalence of Grade 1–4 pneumonitis in Japanese patients is also related to early review and follow‐up chest computed tomography.

The mechanism underlying the development of ALK‐induced pneumonitis is yet to be fully elucidated; however, alveolar epithelial injury is thought to play a role.^52^ Pneumonitis associated with ALK TKIs can be considered as allergic pneumonitis. A low cluster of differentiation (CD)4/CD8 ratio was observed in a large number of lymphocytes (TCD4/TCD8 ratio of 1.4, 90% lymphocytes) in bronchoalveolar lavage fluid collected from patients, which again indicates a hypersensitivity reaction.^41^, ^53^ In addition, ALK TKI‐induced pneumonitis may be associated with an antitumour immune response. Patients with pneumonitis exhibited a positive tumour response. On comparing these individuals to those without pneumonitis, it was found that their PFS was longer (19.9 months and 6.2 months, respectively).^41^

In general, restarting ALK‐targeted therapy in patients with ALK‐induced pneumonitis requires careful consideration. Currently there is a lack of evidence in the guidelines regarding retreatment with ALK TKIs. However, if ALK TKI therapy is stopped due to pneumonitis, the options for follow‐up treatment become extremely limited, which can have a negative impact on patient survival. Nevertheless, there have been many successful cases of ALK TKI retreatment. The safety and efficacy in these patients could be guaranteed even after experiencing Grade 3 or higher pneumonitis.^54^, ^55^ Alternative ALK TKIs are commonly used in practice, as different ALK TKIs may have different mechanisms for the development of pneumonitis.^56^ However, in cases where follow‐up options are limited, some patients have also attempted re‐challenge with ALK TKIs. For example, a 76‐year‐old woman developed severe pneumonitis and respiratory failure a month after initiating alectinib treatment. The patient discontinued alectinib and received mechanical ventilation and methylprednisolone in the intensive care unit. After recovery, a low dose of alectinib was administered. Rechallenge therapy was successful and no pneumonitis occurred after 480 days.^57^ It is important to note that there are also risks associated with restarting ALK TKI therapy. According to another report, a patient who recovered from brigatinib‐induced interstitial lung disease relapsed with pneumonitis immediately after starting lorlatinib treatment.^58^ Further research is needed to determine whether re‐challenging with ALK TKIs carries an increased risk of recurrent pneumonitis. Nevertheless, caution and early prevention measures must be taken when considering this approach in clinical practice. The aetiology of fatal pneumonitis remains largely unknown.

However, several questions remain to be addressed. First, nearly half of the patients with pneumonitis were classified as having high‐grade pneumonitis. Additional retrospective studies are required to elucidate the susceptible population and risk factors for pneumonitis so that clinicians can develop clinical interventions for such patients. Second, given the survival benefits of ALK TKIs, there are currently ongoing clinical trials of ALK TKIs in combination with other agents for treating patients with ALK/ROS1 rearrangements. For instance, in a Phase I/II clinical study of alectinib combined with bevacizumab (NCT03779191), one of 11 patients developed Grade 3 pneumonitis, but the study was terminated early because of slow accrual.^59^ Other trials, such as those of alectinib combined with cobimetinib (NCT03202940), brigatinib combined with platinum‐based chemotherapy (NCT05200481) and brigatinib combined with bevacizumab (NCT04227028), are ongoing. There is a need to further explore the incidence of AEs associated with ALK TKIs in combination with other drug treatments. There are no published data on whether the addition of ALK TKIs can induce high‐grade pneumonitis in patients who received prior radiation therapy.

Our study has some limitations. First, subjective assessments of pneumonitis were performed in the different medical centres and our analysis depended on the quality of the investigators' reports. Heterogeneity was observed among the studies included in this review. In addition, we performed a meta‐analysis at the study level; consequently, patient‐level variables were not available for this analysis. Therefore, we were unable to identify other potential risk factors that may be associated with the development of pneumonitis, including the role of sex, baseline lung function, or smoking history. Larger multicentre RCTs are required to assess the safety of various ALK TKIs in patients with NSCLC.

## 5 CONCLUSIONS

Our study provides more precise data on the incidence of pneumonitis induced by various ALK TKIs. Overall, ALK TKIs had a good pulmonary toxicity profile. Although the incidence of pneumonitis is high, most patients experience mild symptoms and the mortality rate is low after aggressive prevention and treatment. It should be noted that extra caution is needed to prevent the development of pneumonitis in patients being treated with brigatinib, those with a history of prior chemotherapy and those of Japanese ethnicity.

## AUTHOR CONTRIBUTIONS

**Wenting Qie:** Conceptualization (lead); data curation (lead); formal analysis (lead); investigation (lead); methodology (lead). **qian zhao:** Conceptualization (equal); data curation (equal); formal analysis (equal). **Linlin Yang:** Conceptualization (equal); data curation (equal); formal analysis (supporting). **Bing Zou:** Investigation (equal); methodology (equal). **yanan duan:** Formal analysis (supporting); investigation (supporting); methodology (supporting). **Yueyuan Yao:** Investigation (supporting); methodology (supporting). **Linlin Wang:** Conceptualization (equal); formal analysis (equal); funding acquisition (equal); methodology (equal).

## FUNDING INFORMATION

This research was supported by the Natural Science Foundation of Shandong Province (ZR2019LZL012), Jinan Clinical Medical Science and Technology Innovation Plan (202019043), National Natural Science Foundation of China (82172865), The Key Research, Development Program of Shandong (grant number Major Science & Technology Innovation Project) (2021SFGC0501), Start‐up fund of Shandong Cancer Hospital (grant number 2020‐B14) and Clinical Research Special Fund of Wu Jieping Medical Foundation (grant numbers 320.6750.2021‐02‐51 and 320.6750.2021‐17‐13).

## CONFLICT OF INTEREST STATEMENT

The authors declare that they have no conflicts of interest.

## Supporting information


**Data S1.** Supporting Information.

## Data Availability

None.
