# Supplementary material for: Incidence of pneumonitis following the use of different anaplastic lymphoma kinase tyrosine kinase inhibitor regimens: An updated systematic review and meta‐analysis
Source: Cancer Med. 2023 Apr 5;12(13):13873–84. doi: 10.1002/cam4.5913 (PMC10358266; doi:10.1002/cam4.5913)

**Supplementary Table 1. Meta-regression model results for all - grade pneumonitis**

| Variable | Coefficient | SE | 95%CI* | *P* value | Adj R^2^ |
| --- | --- | --- | --- | --- | --- |
| Drug | 0.2204868 | 0.102438 | -0.0008171 – 0.4417907 | 0.2350 | 34.52% |
| Generation | 0.3560065 | 0.0709858 | 0.2047038 – 0.5073093 | 0.2487 | 5.94% |
| Region | 0.3669071 | 0.0686216 | 0.2206436 – 0.5131707 | 0.0942 | 29.48% |
| Study phase | 0.4117724 | 0.0673511 | 0.2682169 – 0.555328 | 0.8627 | -21.00% |
| Treatment line | 0.3233241 | 0.0705632 | 0.1729221 – 0.4737261 | 0.2118 | 21.76% |

Statistically significant at p<0.05.

* Confidence Interval

**Supplementary Table 2. Meta-regression model results for high - grade pneumonitis**

| Variable | Coefficient | SE | 95%CI* | *P* value | Adj R^2^ |
| --- | --- | --- | --- | --- | --- |
| Drug | 0.1436839 | 0.0709976 | -0.0031858 – 0.2905536 | 0.1306 | 18.22% |
| Generation | 0.1431784 | 0.0741406 | -0.0095172 – 0.2958739 | 0.1607 | 50.39% |
| Region | 0.2467587 | 0.0348075 | 0.1750713 – 0.318446 | 0.2408 | 10.08% |
| Study phase | 0.2947694 | 0.034154 | 0.2242789 – 0.3652598 | 0.8249 | -5.94% |
| Treatment line | 0.404758 | 0.0688004 | 0.2630609 – 0.546455 | 0.1729 | 19.21% |

Statistically significant at p<0.05.

* Confidence Interval

**Supplementary Figure 1. The incidences of all-grade dyspnoea and all-grade cough**

**Supplementary Figure 2. The incidence of ≥3 grade dyspnoea**

**Supplementary Figure 3. Funnel plots for (A)all-grade pneumonitis, (B)high-grade pneumonitis, (C)grade 5 pneumonitis**


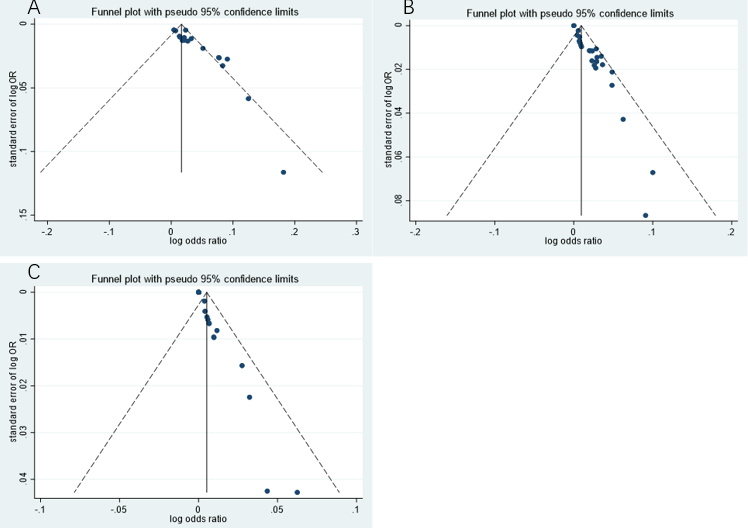

Supplement: Supplementary file 1 — Data S1. Supporting Information. [file CAM4-12-13873-s001.docx]
